# Root-Zone Glyphosate Exposure Adversely Affects Two Ditch Species

**DOI:** 10.3390/biology2041488

**Published:** 2013-12-18

**Authors:** Lyndsay E. Saunders, Melissa B. Koontz, Reza Pezeshki

**Affiliations:** Department of Biological Sciences, University of Memphis, Memphis, TN 38152, USA; E-Mails: mlee1@memphis.edu (M.B.K.); pezeshki@memphis.edu (R.P.)

**Keywords:** agricultural runoff, glyphosate, drainage ditches, phytotoxicity, non-target species, wetland plants

## Abstract

Glyphosate, one of the most applied herbicides globally, has been extensively studied for its effects on non-target organisms. In the field, following precipitation, glyphosate runs off into agricultural ditches where it infiltrates into the soil and thus may encounter the roots of vegetation. These edge-of-field ditches share many characteristics with wetlands, including the ability to reduce loads of anthropogenic chemicals through uptake, transformation, and retention. Different species within the ditches may have a differential sensitivity to exposure of the root zone to glyphosate, contributing to patterns of abundance of ruderal species. The present laboratory experiment investigated whether two species commonly found in agricultural ditches in southcentral United States were affected by root zone glyphosate in a dose-dependent manner, with the objective of identifying a sublethal concentration threshold. The root zone of individuals of *Polygonum hydropiperoides* and *Panicum hemitomon* were exposed to four concentrations of glyphosate. Leaf chlorophyll content was measured, and the ratio of aboveground biomass to belowground biomass and survival were quantified. The findings from this study showed that root zone glyphosate exposure negatively affected both species including dose-dependent reductions in chlorophyll content. *P. hydropiperdoides* showed the greatest negative response, with decreased belowground biomass allocation and total mortality at the highest concentrations tested.

## 1. Introduction

Glyphosate is one of the world’s most widely used herbicides [1,2], and its use has greatly increased over time, with amounts applied in the United States doubling in half a decade, from 41,000-t in 2001 to 84,000-t in 2007 [3]. Glyphosate is applied to agricultural fields at the beginning of the growing season to eliminate undesirable vegetation before planting with crops. Additionally, many fields are planted with glyphosate-resistant crop varieties and receive repeated glyphosate applications throughout the growing season.

The negative effects of foliar exposure on non-target vegetation through drift or by-spray are well-documented in the literature [4,5,6]. However, a less investigated exposure pathway occurs when non-target vegetation in edge-of-field ditches is exposed to aqueous glyphosate in the root zone following soil infiltration during precipitation events [1,7]. Within a glyphosate exposed plant, the enzymatic action of the shikimate pathway is inhibited, and chorismate, the end product of this pathway, can no longer be produced. Chorismate is the precursor molecule for the aromatic amino acids, phenylalanine, tryptophan, and tyrosine, and for a variety of essential secondary metabolites. Chorismate-derived compounds are then utilized by the plant in numerous functions contributing to growth and reproduction [8,9]. In addition, the cascade of effects following glyphosate exposure decreases chlorophyll content in plants [10]. As a water-soluble compound, glyphosate is found in runoff from agricultural fields that enters ditches, canals, and receiving surface waters [1], where it has an aquatic half-life of 7–14 d [1,11]. This exposure may influence species composition of agricultural drainage ditches and, in turn, affect the ecological services ditches perform [12].

The objective of this experiment was to test a range of exposure concentrations to assess a threshold for sublethal root-zone glyphosate exposure. The exposure concentrations used were chosen to represent a single acute exposure event. The two highest concentrations used, 1,000 and 10,000 mg/L, are of the same magnitude as those obtained following label preparation instructions for a commercial product containing glyphosate which is used for agriculture, as well as non-agricultural applications, such as habitat management, parks, residential areas, and roadsides. The label preparation instructions direct a preparation of 1.1% product solution which contains 8,310 mg/L glyphosate (corresponding to the 10,000 mg/L exposure), while preparation of a 0.3% product solution would contain 2,210 mg/L glyphosate (corresponding to the 1,000 mg/L exposure). Label instructions can be followed to prepare up to an 11.1% product solution, which would contain 82,020 mg/L glyphosate. Additionally, the highest concentration of 10,000 mg/L is of the same magnitude as the Expected Environmental Concentration (ECC) value of 42,840 mg/L calculated for non-target terrestrial plants inhabiting semi-aquatic low-lying areas for runoff following aerial application using formulae from the USEPA [13]. The low concentration assessed (10 mg/L) was chosen to examine an intermediate exposure. We predicted that increasing root zone glyphosate exposure concentrations would be associated with negative plant responses for two ruderal species, *Polygonum hydropiperoides* and *Panicum hemitomon*, commonly found in agricultural ditches in southcentral United States. Specifically, we tested the hypotheses that both species would exhibit dose-dependent reductions in chlorophyll content index, root-to-shoot ratios, and survival.

## 2. Materials and Methods

### 2.1. Plant Material

Two wetland species commonly found in agriculture ditches were selected for study: *Polygonum hydropiperoides* and *Panicum hemitomon*. *Polygonum* spp. (smartweed), an erect perennial forb in the family Polygonaceae, was present in 100% of the smallest ditch class in surveyed agricultural drainage ditches in the Mississippi Delta region [14,15]. *P. hemitomon* (maidencane), an erect perennial graminoid in the family Poaceae with a C_3_ photosynthetic pathway, is also a species commonly found in ditches [16]. In Tennessee, however, it is listed as a species of Special Concern for its protection status. Both species are wetland obligates with distributions that include the Mississippi Delta [15]. Plants were collected from wild populations in wetland cells maintained at the USDA NRCS Jamie L. Whitten Plant Materials Center in Coffeeville, Mississippi (N 33°59'20.875", W 89°47'28.925").

### 2.2. Experimental Procedures

Following collection, plants were standardized by cutting individuals to 15 cm stem and 10 cm root and then were potted in PVC pots (60 cm h × 5 cm d) containing washed commercial play sand, limiting the adsorption of glyphosate onto organic matter [17]. Plants were maintained for 4 weeks in a climate-controlled greenhouse (20–31 °C) at the University of Memphis without supplemental lighting. Plants were watered daily with tap water and received weekly fertilizer applications at a rate of 1.25 g/L 20-20-20 Peter’s fertilizer (Scotts MiracleGrow Company, Marysville, OH, USA). Following the maintenance period, individuals were transferred to a laboratory equipped with supplemental light on a 16-h photoperiod, illuminated by four 400 W high pressure sodium and four 400 W metal halide lamps in water-cooled ballasts, providing approximately 1,000 µmol·m^−2^·s^−1^ photosynthetic photon flux density at the leaf canopy level. The study was initiated after a seven day acclimation period in the laboratory and was terminated 21 d after glyphosate exposure.

*Polygonum hydropiperoides* and *Panicum hemitomon* and four glyphosate concentrations (0, 10, 1,000, 10,000 mg/L glyphosate) were arranged in a 2 × 4 randomized block design. Exposure solutions were prepared using deionized water and the commercial product Roundup ProDry (EPA Registration No. 524-505) which contains 71.4% glyphosate in the form of an ammonium salt of *N*-(phosphonomethyl)glycine and 28.6% other ingredients (Monsanto Company, St. Louis, MO, USA). During exposure, 100 mL glyphosate solution of the appropriate concentration was introduced to the top of the substrate and allowed to infiltrate for two hours, after which the substrate was rinsed with 500 mL deionized water.

### 2.3. Plant Measurements

Leaf chlorophyll content index (CCI) was recorded prior to treatment initiation and daily thereafter for the study duration using a chlorophyll content meter (CCM-200, Opti-Sciences, Tyngsboro, MA, USA). Measurements were obtained from the third fully expanded leaf from the top of the apical stem. Following the study termination on day 21, plants were divided into above- and below-ground tissue and dried in an oven at 70 °C until a constant weight was reached, then the dry weights were recorded. These dry weights were used to calculate the ratio of aboveground biomass to belowground biomass. Survivorship was also calculated.

### 2.4. Data Analyses

Blocking of glyphosate treatments by species was required to minimize shading introduced by the species’ different growth habits. Due to limited laboratory space, each glyphosate exposure treatment was replicated by six *Polygonum hemitomon* plants (N = 24) and 10 *Panicum hydropiperoides* plants (N = 40). Differences in means for pre-exposure and post-exposure CCI were analyzed using a repeated measures analysis of variance (ANOVA) with two sampling dates and four levels of glyphosate treatment as independent factors. Differences in means for root:shoot ratios and for differences among treatment groups for survival for each species were analyzed for each species using a one-way ANOVA with four levels of glyphosate exposure as the independent factor [18]. Significant differences were followed by a Tukey’s post-hoc comparison. Differences were considered significant at α < 0.05.

## 3. Results

### 3.1. Chlorophyll Content Index

Analysis of pre- and post-exposure CCI measurements showed a significant interactive effect and significant time effect for *Polygonum hydropiperoides* (time*treatment: F_3,39_ = 8.646, *p* < 0.001; time: F_3,39_ = 85.171, *p* < 0.001) and *Panicum hemitomon* (time*treatment: F_3,19_ = 5.525, *p* < 0.01; time: F_3,19_ = 14.727, *p* = 0.001).

**Figure 1:**
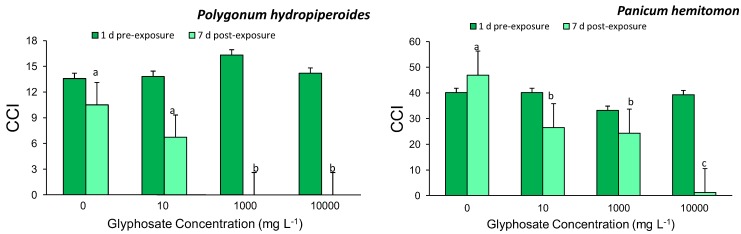
Leaf chlorophyll content index (CCI) values for *Polygonum hydropiperoides* and *Panicum hemitomon*. Bars represent treatment means ± SE for 10 and six replicates for *P. hydropiperoides* and *P. hemitomon*, respectively. Dark bars represent 1 d pre-exposure values; light bars represent 7 d post-exposure values. Lowercase letters represent significant differences across glyphosate treatments for species (*p* < 0.05). Note the different scales for CCI for each species.

In both *Polygonum hydropiperoides* and *Panicum hemitomon*, CCI did not differ among treatments before exposure. For both species, plants exposed to root zone glyphosate had significant decreases in CCI after seven days. In *P. hydropiperoides*, CCI after seven days decreased with increasing glyphosate concentration and resulted in mortality for the 1,000 and 10,000 mg/L treatments. *P. hemitomon* also exhibited a dose-dependent reduction in CCI values after seven days (Figure 1).

### 3.2. Root-to-Shoot Biomass Ratios

**Figure 2:**
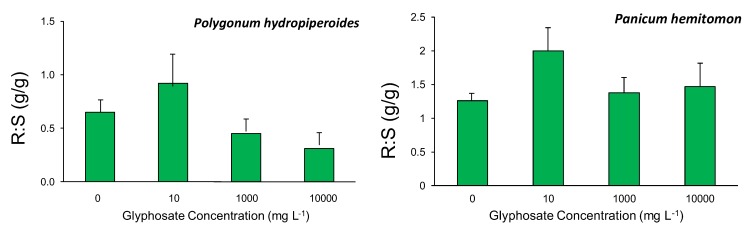
Root-to-shoot biomass ratios (R:S, g/g) for *Polygonum hydropiperoides* and *Panicum hemitomon*. Bars represent treatment means ± SE for 10 and six replicates for *P. hydropiperoides* and *P. hemitomon*, respectively.

Root-to-shoot biomass ratios (R:S) were not affected by glyphosate exposure treatments in *Polygonum hydropiperoides* (F_3,39_ = 2.46, *p* = 0.077) or *Panicum hemitomon* (F_3,19_ = 1.91, *p* = 0.162). Trends of resource allocation, however, while not statistically significant, differed between the two species, as shown in Figure 2. In *P. hydropiperoides*, all treatments showed a greater allocation to shoot biomass as compared to root biomass, with a trend of decreasing root allocation with increasing glyphosate exposure concentration. In *P. hemitomon*, all treatments showed a greater allocation to shoot biomass as compared to root biomass, with little variation among treatments.

### 3.3. Survival

**Figure 3:**
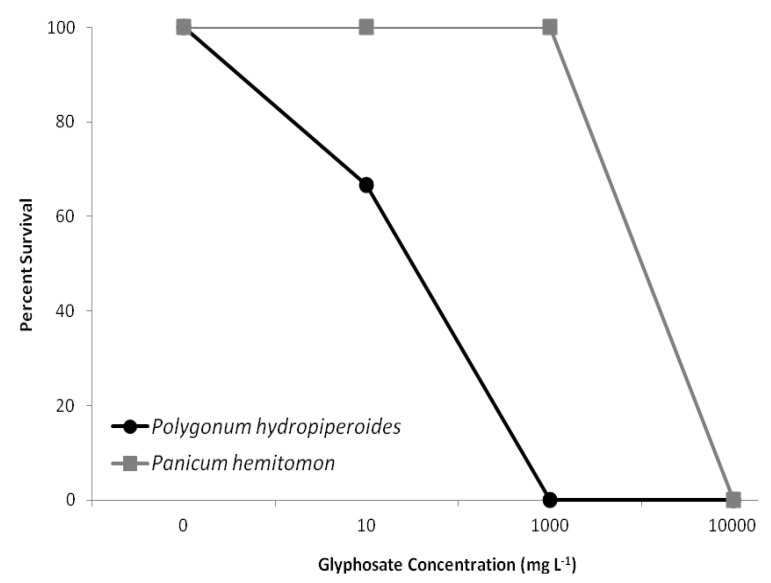
Interactive effects of species and glyphosate concentration on survival.

Glyphosate exposure affected survival in *Polygonum hydropiperoides* and *Panicum hemitomon* (F_3,58_ = 14.508, *p* < 0.001). Each species showed a different rate of survival during the experiment duration (F_1,58_ = 8.825, *p* = 0.005). An interactive effect between species and glyphosate exposure also affected survival (F_3,58_ = 9.733, *p* < 0.001) (Figure 3). Survival decreased with increasing glyphosate concentration, with total mortality seen in the 1,000 and 10,000 mg/L treatments for *P. hydropiperoides* (Figure 3). In *P. hemitomon*, 100% survival was seen in the 0, 10, and 1,000 mg/L treatments and total mortality observed in the 10,000 mg/L treatment (Figure 3).

## 4. Discussion and Conclusions

Exposure of two common agricultural ditch plants, *Polygonum hydropiperoides* and *Panicum hemitomon*, to different concentrations of glyphosate in the root zone resulted in significant decreases in CCI and survival while root:shoot ratios were unaffected. The study was successful in identifying a sublethal glyphosate root zone exposure concentration for both species. These findings partially support our prediction that increasing root zone glyphosate exposure concentrations would result in negative plant responses.

Studies have experimentally confirmed plants’ ability to take up glyphosate following root exposure and translocate the compound to other tissues. Alister *et al*. showed that 14^C^-glyphosate is taken up through the roots of *Zea mays* L. seedlings and transported to other tissues, particularly the shoot apex [19]. Glyphosate is accumulated in the greatest proportion in the meristematic tissues, affecting developing tissues most directly [9]. Chlorophyll turnover is known to be dynamic, with synthesis and degradation occurring in durations ranging from minutes to days [20]. These studies support our finding that root zone glyphosate exposure adversely affects leaf chlorophyll content, thus the observed decreases in CCI, with increasing intensity of exposure.

Perennial grasses have been found to accumulate glyphosate in the rhizomes and stolons [21]. Furthermore, up to 35% of whole plant dry biomass can be accounted for through processes requiring chorismate, the essential molecule whose synthesis is inhibited by glyphosate exposure [9]. The lack of significant differences in root:shoot ratios among different treatments was unexpected based on these previous studies. The short duration of the experiment was required due to extensive mortality at the higher glyphosate concentrations, but 21 d may not have been a sufficient amount of time to get significant differences in biomass partitioning.

The low survival rate for *Polygonum hydropiperoides* exposed to the highest two glyphosate exposure concentrations was predictable given that these two dosages are of the same magnitude of solutions prepared following packaging instructions (1,000 and 10,000 mg/L). *Panicum hemitomon* was able to survive with no mortality for all treatments except for the highest concentration. The differential survival rates between *P. hemitomon* and *P.hydropiperoides* was the least expected and most interesting finding from this study. The ability of *P. hemitomon* to survive a broad range of glyphosate concentrations in its root zone may contribute to its relative abundance in ditches adjacent to agricultural fields.

These data may have important implications for management of agricultural ditches to provide maximum ecological benefits. Plant coverage was identified as the most important factor affecting pesticide removal from ditches in a recent literature review [22]. Syversen and Bechmann demonstrated that grass buffer zones remove up to 48% of the herbicide glyphosate present in surface runoff experiments [23]. As a grass species, *Panicum hemitomon* may be well-suited to plant assemblages that are subject to exposure to glyphosate. It is important to note, however, that the present study was conducted in sand, a soil texture not representative of most field conditions, a limitation that should be considered in implementation of best management practices based on these findings.

Root zone glyphosate exposure is an under-investigated pathway with important ecological implications. These findings highlight how the root-zone exposure pathway differentially affects non-target vegetation. To elucidate the dynamics of root-zone glyphosate exposure in plants, experimental approaches that explore variables such as exposure duration and inter-specific interactions within plant assemblages will be especially illuminating. This experiment demonstrates the interesting work that results from investigating root-zone glyphosate exposure.
